# A cross-sectional sero-survey on preoperative HBV vaccination policy in Poland

**DOI:** 10.1186/s12879-017-2607-2

**Published:** 2017-07-25

**Authors:** Maria Ganczak, Marcin Korzen, Alina Jurewicz, Zbigniew Szych

**Affiliations:** 1 Department of Epidemiology and Management, Pomeranian Medical University, Zolnierska 48, 71-210 Szczecin, Poland; 2 Department of Methods of Artificial Intelligence and Applied Mathematics, West Pomeranian University of Technology, Zolnierska 49, 71-210 Szczecin, Poland; 3 Department of Orthopedic and Trauma, Pomeranian Medical University, Unii Lubelskiej 1, 71-210 Szczecin, Poland; 4 Department of Computer Science and Education Quality Research, Pomeranian Medical University, Zolnierska 53, 71-210 Szczecin, Poland

**Keywords:** Preoperative, Vaccination, Hbv, Schedule, Anti-HBs, Protection

## Abstract

**Background:**

A two-dose preoperative vaccination schedule against HBV has been the widely accepted policy in Poland. However, its effectiveness has not yet been assessed.

Objective: To evaluate a two-dose preoperative HBV vaccination policy by an assessment of the proportion of patients who don’t present a protective level of anti-HBs (<10.0 mIU/ml).

**Methods:**

Consecutive patients from surgical/gynecologic wards of 12 randomly selected hospitals in West Pomerania, Poland, hospitalized between 2010 and 2013, vaccinated against HBV with a two-dose regimen, were asked to complete an anonymous questionnaire. Serum samples were assayed for anti-HBs with the use of third-generation testing methods. To compare sensitivity versus specificity across a range of values for the ability to predict a dichotomous outcome (a protection against HBV infection) a Receiver operating characteristic (ROC) curve was determined.

**Results:**

There were 193 patients, 58.5% women, median age 52 years. Almost a half (46.0%) of the patients were operated on within 0–60 days of taking the second vaccine dose, 16.2% - 61-180 days after, 37.8% >180 days after. Anti-HBs titer was below a protective level in 49.2% of participants (0.0 mIU/ml in 17.8%, 0.1–9.9 mIU/ml in 31.4%); none of them were aware of this fact. Age ≤ 52 years (OR = 1.89) and having surgery more than 37.5 days after HBV vaccination (OR = 2.70) were associated with greater odds of being protected against HBV infection through vaccination. For the time frame between the second dose implementation and surgery 23 days, a sensitivity of 84% and specificity of 22% for obtaining protection against HBV infection was found, for the time frame >37.5 days – sensitivity remained high (80%), while specificity increased (41%); there was an apparent peek on the ROC curve between 38 and 60 day. In the group vaccinated 0–37.5 days before surgery, less patients had the protective level of anti-HBs titer than in vaccinated 38–60 days before surgery (32.3% vs 60.0%; *p* = 0.03).

**Conclusions:**

The success rate in achieving adequate immune protection with two dose HBV vaccination schedule in preoperatively vaccinated patients is relatively low, especially among those vaccinated less than five weeks prior to surgery. In more than a third of cases the standard three-dose regimen could have been implemented, as participants had time to complete a third dose. Current recommendations regarding a preoperative policy with a 2-dose vaccination schedule in Poland should be revised; the best time to perform surgery after the implementation of the second dose of vaccine in the context of patient protection against HBV infection would be between 38 and 60 days.

## Background

The introduction of a vaccine, which is now one of the most widely used worldwide, has reduced the incidence of hepatitis B virus (HBV) infections, especially for risk groups [1, 2].

Although Poland has a relatively low prevalence of hepatitis B surface antigen (HBsAg) carriers (1.5%) in the general population, it is noteworthy that, according to data from 1970s–1990s, over 50% of total hepatitis B cases and about 90% of cases in patients over 60 years of age were nosocomially acquired [3, 4].

To reduce infections generated in health care facilities, active immunisation against HBV was required from elective surgery patients between 1993 and 1997 [3, 4]. This was included in the National Immunization Program (NIP), together with neonatal vaccination against HBV, vaccination of recipients of blood and blood products, hemodialysed patients, household members and the sexual partners of HBsAg carriers, as well as health care workers and medical students [5, 6]. Regarding elective surgery patients, the vaccination schedule was complementary to the course recommended for other vulnerable groups, i.e. 0–1-6 months; *Engerix* or *Hepavax*, 20 mcg per dose, were used [6]. According to reports from 1997, 681,000 patients were immunized due to this regulation [5]. The suspension of this directive in 1997 has been due to the improvements in aseptic conditions, and an increase in disposable medical device use [3]. Another explanation was the result of a debate with the involvement of experts in the field, where the opponents argued that pre-operative HBV vaccination policy might be a stimulus for facilities to neglect infection control procedures [5].

Although the immunisation requirement for preoperative HBV vaccination no longer applies, currently this procedure is still recommended by the NIP [6] and an immunisation certificate is still unofficially required for elective surgical procedures by a number of healthcare facilities. This might be due to the fact that, though dramatic, a 10-fold decrease in HBV incidence has been observed in the last 35 years (from around 15,000 in 1978 to 2457 in 2014), medical procedures are still reported as the most common route of HBV transmission in Poland [7, 8]. It has been established that in 2014 the majority of acute and chronic infections (75% and 65% respectively) were most probably associated with medical procedures accompanied by skin breakdown [8]; aforesaid high percentages have not been observed in other developed countries.

A scrupulous preoperative vaccination policy may influence the high HBV immunisation coverage among surgical patients, especially regarding elective procedures [9]. Evidence shows that patients follow the recommendations of referring surgeons and immunise themselves against HBV before the operation; thus – from a public health view point - this policy seems to be an effective tool to limit the spread of the epidemic in Poland [9]. The HBV vaccination rates among Polish patients range between 54% to 60% [8–11], much higher uptake than reported by some other authors from abroad (26%–33%) [12–15].

However, it has always been common practice in Poland for elective surgery to be carried out on patients two weeks after the second dose of HBV vaccine was administered. Previous studies have shown that the immune protection after taking two doses is not satisfactory and insufficient [16–22]. Interestingly, despite the universal use of the preoperative immunisation policy in Poland since 1993, the extent to which operated patients are protected against HBV infection has never been evaluated.

Therefore, the objective of this study was to evaluate the preoperative immunisation policy in Poland by assessing the fraction of surgical and gynecological patients, vaccinated preoperatively against HBV with 2 doses of vaccine, and presenting a protective level of anti-HBs during surgery.

## Methods

### Design & setting

A cross-sectional sero-epidemiological survey was conducted between 2010 and 2013.

### Study population & sampling

The study population consisted of adult consecutive patients from surgical/gynecological wards of 12 randomly selected hospitals in the Western Pomerania region, Poland. The following eligibility criteria needed to be met in order to participate in the study: to be at or over 18 years of age, ability to give informed consent, admission for an elective procedure, previous vaccination against HBV only with a 2-dose regimen, and patient agreement to take part in the study. The patient interviews were conducted after they were admitted to the relevant wards. Patients were asked to give a serum sample to assess anti-HBs levels. Those with evidence of a current/previous HBV infection were excluded; this was assessed on the base of a self-assessment in the questionnaire and by anti-HBc test which had been carried out by a patient prior to the admission, or at the ward, after admission. HBV immunization status was based on self-reports of previous immunization and on the results of copies of vaccination cards delivered by patients on admission.

The sampling frames included a list of hospitals in West Pomeranian region of Poland obtained from the local health department. Only hospitals which comprised of surgical, gynecological, pediatric and internal medicine wards were included. Stratified sampling was used. Firstly, hospitals were stratified into urban hospitals from the city of Szczecin (the capital of the region with 405,606 inhabitants) and provincial hospitals from all 16 districts in the region, to ensure representation of different practice levels, with random selection of a half (*n* = 3) of urban hospitals from the capital of the region and a half (*n* = 8) of provincial hospitals from the list. In the next step, a random sampling of one surgical and one gynecological ward was made for hospitals with more than one surgical/gynecological ward. A code was given to each patient, for the questionnaire and for the blood sample. At each selected hospital blood samples were collected within a two month period from all eligible patients who fulfilled the inclusion criteria.

### Study instrument

A questionnaire administered by trained nurses included questions that queried patients on the following:

- demographic, including age, gender, weight, height, place of residence (with possible options: “city of Szczecin”, “other town”, “village”)
- the name/type of the hospital to which they were admitted (possible options: “urban” for hospitals located in the city of Szczecin, the capital of the region, and “provincial” – for hospitals located in districts around the West Pomeranian region)
- reasons for HBV vaccination (possible options: “a request of the referring surgeon/ gynecologist”, “a request of the family doctor”, “a recommendation of a friend/family member”, “media campaign” and “other reason”)
- the interval between the first dose and second dose of HBV vaccine (days), the date of the second dose implementation, the date of admission for an elective procedure
- risk factors regarding immunological response to vaccination: smoking, co-morbidities impairing a correct response (diabetes, liver disease), reported dialysis or immuno-supression
- being informed about the mechanisms of HBV protection via immunization by a doctor referring for vaccination (“yes”, “no”)
- being asked to be tested for anti-HBs level 1–2 months after immunization (“yes”, “no”)

### Sero-testing

Enzyme-linked immunosorbent assay (ELISA) system version 3.0 was used to detect anti-HBs (Hoffman-La Roche Ltd., Basel, Switzerland). Testing was performed in one referential laboratory in the teaching hospital in Szczecin. Two weeks after sampling the participants could call the investigators at a dedicated phone line and obtain their results by stating their code.

### Statistical analysis

Data were validated using a customized program STATISTICA PL Version 7.1. (StatSoft Inc., 2012) and R (R version 3.x) software [23]. Continuous variables were expressed as mean and standard deviation or median and minimum–maximum values; categorical variables were expressed as frequencies and percentages. Our primary outcome variable was protection after HBV vaccination (anti-HBs level ≥ 10 mIU/ml) and we aimed to identify variables associated with this outcome. Bivariate analysis assessed demographic characteristics: age (below/above the median, i.e. 52 years), gender, BMI (≤25/>25 kg/m^2^), together with risk factors impairing immunological response to vaccination: smoking, co-morbidities impairing a correct response (diabetes, liver disease, renal insufficiency) or immunosuppression (yes/no) and time from HBV vaccination to the surgery (days), associated with an outcome variable. For categorical variable groups were compared using the chi-square test with Yates’ correction and Fisher’s exact test, whilst the U Mann-Whitney test was used for numeric variables to identify the bivariate impacts on immune response. *P* value of <0.05 was set for statistical significance. To build a logistic regression model [24] a set of predictors was used. The final associations between predictors and the outcome variable were measured with the use of coefficients of a logistic regression model. Coefficients for binary variables are equal to the natural logarithm of the odds ratio; OR = exp.(beta).

In addition, to compare sensitivity versus specificity across a range of values for the ability to predict a dichotomous outcome (a protection against HBV infection) a Receiver operating characteristic (ROC) curve [25] was determined based on 193 observations. The area under the ROC curve was calculated to measure test performance.

## Results

### Patient characteristics

Among 236 patients invited to participate, 203 agreed (86.0%), of whom 10 were excluded due to a previous HBV infection. There were 193 participants (median of age 52 years), 58.5% females. Most of the participants (81.7%) lived in urban areas, 18.3% - in rural areas. The mean BMI was 26.73 ± 4.52 kg/m^2^. Smoking at the time of vaccination was reported by 24.9% of participants, 13.5% reported having co-morbidities: diabetes (*n* = 18), hepatitis C (*n* = 4), renal insufficiency (*n* = 2) and being on immunosupression (*n* = 2). Almost three fourths of participants (*n* = 143, 74.1%) were from the provincial hospitals, the rest – from the urban hospitals.

### Reasons for HBV vaccination

Out of 193 participants, 180 gave reasons for immunization: 82.2% (*n* = 148) were immunized due to the recommendation of referring surgeons, 7.2% (*n* = 13) due to the recommendations of family doctors, 3.3% (*n* = 6) due to media campaigns, 1.1% (*n* = 2) due to the recommendations of family/friends, the rest (6.1%; *n* = 11) was vaccinated for other reasons.

None of 148 participants immunized preoperatively due to the recommendation of referring surgeons or general practitioners were informed about the mechanisms of HBV protection via immunization, none were asked to test for anti-HBs level 1–2 months after immunization.

### Interval between the first and the second dose

Patients were asked about the interval between the first and the second dose of HBV vaccine; the range was 29–43 days, the median: 32 days. The vast majority of patients (*n* = 172, 89.1%) took the second dose 30–33 days after taking the first dose.

### Response rates to HBV vaccination

Regarding time between taking the second vaccine dose and an operation, 86 (44.6%) of the patients were operated on within 0–60 days after taking the second dose, 30 (15.5%) - 61-180 days after, and 77 (39.9%) >180 days after (range 180–6935). There were no differences in age (*p* > 0.93), gender (*p* > 0.28), BMI (*p* > 0.34), smoking habit (*p* > 0.20) and co-morbidities (*p* > 0.73) between the 3 sub-groups.

**Fig. 1:**
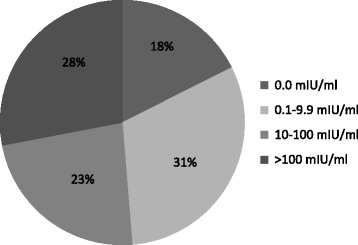
Preoperative patients anti-HBs levels after receiving 2 doses of HBV vaccine. West Pomerania, Poland; 2010–13

The number of patients with inadequate immune protection (anti-HBs < 10mIU/ml) was 94 (48.7%); anti-HBs titer was 0.0 mIU/ml in 17.6% patients, 0.1–9.9 mIU/ml - in 31.1%. In 45 patients (23.3%) anti-HBs titer was 10.0–100.0 mIU/ml, in 54 (28.0%) it was >100 mIU/ml (Fig. 1).

The median anti-HBs titer in all patients vaccinated with 2 doses was 12.6 mIU/ml (range 0–2543.0). In patients with an adequate immune protection it was 127.2 mIU/ml (range 10.6–2543.0), in those with an inadequate protection – 1.25 mIU/ml (range 0.0–9.8); *p* = 0.0001. Patients with an adequate protection were younger than those with the inadequate (age: $\overline{\mathrm{x}}$=48.1 ± 14.9 vs $\overline{\mathrm{x}}$=52.3 ± 13.7 years respectively); *p* = 0.04. There were no differences in gender (*p* = 0.11), BMI (*p* = 0.16), smoking habit (*p* = 0.97) and co-morbidities (*p* = 0.50) between the 2 sub-groups.

All variables were then entered into a logistic regression model. It revealed that older age (odds ratio (OR) = 0.53) was associated with lower odds of being protected against HBV infection through vaccination and having surgery more than 37.5 days after HBV vaccination (OR = 2.70) was associated with greater odds of being protected (Table 1).

**Table 1:** Logistic regression model: association of protection from vaccination against HBV with selected variables (OR estimates and 95%CIs), *n* = 193; AUC = 0.671

Variable	OR	95% CI
Age: >52 years	0.53	0.29–0.97
Gender: male	0.79	0.42–1.49
BMI: >25 kg/m^2^	0.80	0.41–1.55
Smoking: yes	1.09	0.55–2.22
Co-morbidities impairing a correct response^a^/ immunosupression: yes	1.34	0.48–3.98
Time from HBV vaccination to the surgery: >37.5 days	2.70	1.40–5.32

^a^diabetes, hepatitis C, renal insufficiency

**Fig. 2:**
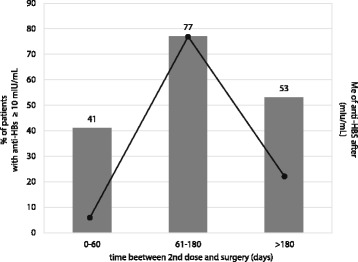
Percentage of patients operated on with protective level of anti-HBs and medians of anti-HBs titers by time between receiving 2 doses of HBV vaccine and surgery

In those vaccinated 0–60 days before surgery, anti-HBs titer <10.0 mIU/ml was presented significantly more often (*n* = 51; 59.3%) compared to those vaccinated 61–180 days before (*n* = 7; 23.3%), *p* = 0.0007; Fig. 2. Significantly less patients vaccinated 61–180 days before surgery presented anti-HBs titer <10.0mIU/ml, compared to those vaccinated >180 days (*n* = 7; 23.3% vs *n* = 36; 46.8%), *p* = 0.03. There was no statistically significant difference between the percentages of patients presenting anti-HBs titer <10.0 mIU/ml and vaccinated 0–60 days before surgery and those vaccinated >180 days before (59.3% vs 46.8%), *p* = 0.12.

The median anti-HBs titer in patients vaccinated 0–60 days before surgery was lower than was in those vaccinated 61–180 days before (6.05 mIU/ml, range 0–2543.0, and 77.20 mIU/ml, range 0–1001.1, respectively); *p* = 0.0004 but it did not differ significantly from the median observed in patients vaccinated > 180 days before (22.9 mIU/ml, range 0–1022.0); *p* = 0.18. The median anti-HBs titer in patients vaccinated 61–180 days before the surgery was higher than observed in those vaccinated >180 days before; *p* = 0.01, Fig. 2.

### Sensitivity and specificity regarding time between the implementation of the second dose of HBV vaccine and surgery

As presented in Table 2, for the time frame between the second dose implementation and surgery 23 days, a sensitivity of 84% and specificity of 22% for obtaining protection against HBV infection was found. For the time frame >37.5 days – sensitivity remained high (80%), while specificity increased (41%).

**Table 2:** Sensitivity and specificity regarding time between the implementation of the second dose of HBV vaccine and surgery in the context of obtaining protection against HBV infection

Time^a^ (days)	Sensitivity	Specificity	OR	CI
>23	0.84	0.22	1.49	0.68–3.30
>37.5	0.80	0.41	2.79	1.41–5.62
>59.5	0.66	0.54	2.26	1.22–4.22
>178	0.41	0.62	1.14	0.61–2.11

^a^between second dose implementation and surgery

### Receiver operating characteristic (ROC) curve

**Fig. 3:**
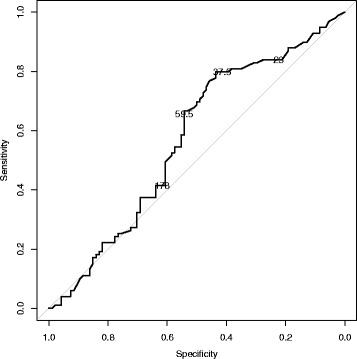
A ROC curve for various cut-off levels of days between vaccination (2 doses) and surgery in differentiating between protection from HBV infection and the lack of protection

The ROC curve for the data based on 193 observations has the form presented at Fig. 3.

In the ROC curve for the data based on 193 observations (Fig. 3), days between (DB) the implementation of the second dose of HBV vaccine and surgery are plotted for their ability to predict protection against HBV infection (a dichotomous outcome) with true positives on the vertical axis (sensitivity) and false-positives (specificity) on the horizontal axis. At lower DB cut-offs, e.g. 23 days, there is higher sensitivity or better ability to predict protection, although this is compromised by lower specificity (i.e. the test falsely identifies more subjects without protection). In these cases the anti-HBs concentration of ≥10 mIU/ml seems to be random, and there is an apparent peek on the ROC curve between 38 and 60 day regarding DB the implementation of the second dose of HBV vaccine and surgery.

In the group vaccinated 0–37.5 days before surgery less patients had the protective level of anti-HBs titer than in vaccinated 38–60 days before surgery (20/62; 32.3% vs 15/25; 60.0%; *p* = 0.03).

## Discussion

### Results overview

To our knowledge, this is the first study which had focused on an evaluation of the preoperative HBV immunisation policy in Poland with 2 doses of vaccine, regarding its possible effect on protection against acquiring a nosocomial HBV infection. Among patients who were preoperatively vaccinated with two doses, anti-HBs titer was below a protective level in almost a half, none of those were aware of this fact. There was an apparent peek on the ROC curve between 38 and 60 day regarding days between the implementation of the second dose of HBV vaccine and surgery. In the group vaccinated up to 37.5 days before surgery significantly less patients had the protective level of anti-HBs titer than in vaccinated 38–60 days before surgery. In more than a third of cases the standard three-dose regimen could have been implemented, as participants had time to complete a third dose.

### Preoperative vaccination policy in patients vaccinated before surgery with a two-dose schedule in the context of anti-hepatitis B immunity status

Results of our previous study, conducted in 2009, showed that, even as there are no requirements or standard protocols for preoperative HBV immunisation in Poland, many surgeons still implement their own program: regarding elective procedures, 82% of surgical patients were vaccinated for HBV preoperatively, almost two thirds of whom were immunized at the request of referring surgeons; about one third of those vaccinated patients was immunized with a 2-dose vaccination schedule [9]. Another study, conducted by us in 2013 among patients attending a primary care clinic, revealed that planned elective surgery was the main reason for their HBV immunization; similar rates were observed among adult patients from the Polish city of Katowice [10, 11].

As planned elective surgery is the main reason for HBV vaccination regarding non-immunized adults and the preoperative immunization policy positively influences the HBV vaccination uptake in Poland, it should not be discontinued.

However, the results of the present survey reveal that this policy only partly fulfills its main goal - to reduce infections generated in health care facilities, since the regimen does not protect a significant fraction of patients against HBV infection. It refers especially to those vaccinated less than two months before surgery: overall almost one in two of all study participants who took two doses of HBV vaccine were found to present anti-HBs titer below protective level before operation, but almost two in three of those vaccinated 0–60 days before they were operated on.

Our findings are in line with the other studies [16–19]; first of those were carried out in 1980s–1990s [20–22]. As an example, 30% of infants, surveyed in 1984 in Senegal, and 11–27% of medical staff surveyed independently in Poland (1991) and Israel (1993) showed a lack of immune response to HBV vaccination after taking two doses of HBV vaccine [20–22]. More recently, a lack of sero-positivity after two doses of HBV vaccine was found in 51% of adult Indian patients, in 11% of medical staff in Ceylon and in 8% of Chinese college students [16–18]. Regardless of geographical region, time or population group surveyed, a significant proportion of vaccinated individuals showed a lack of protection against HBV infection after taking two doses of vaccine.

In our preliminary study on participants vaccinated before surgery, who took a 3-dose vaccination schedule, 88% presented protective levels of anti-HBs; significantly more than those who took only two doses [19]. This is supported by others: protection was obtained in 86–99% of vaccinated individuals after taking a third dose of HBV vaccine [16–18, 20–22].

According to the results of this study, it may be concluded that HBV immunization with a 2-dose schedule induces slow and weaker immunological response when compared with a 3-dose schedule. After taking two doses only, the highest percentage of protected patients was observed in the group operated on 60–180 days after immunization, not in the group operated on up to 60 days. The percentage of protected patients operated on over 180 days after immunization was similar to observed in the group operated on up to 60 days. Regarding the peak level of the anti-HBs after taking the third dose, Honorati et al. and found that it was reached 68 days after and remained stable for several years [26]. Piratheepkumar et al. found that after two doses of vaccination, there was significant deterioration in protective immunity after four years. However, in individuals who received three doses - the protective immunity did not reduce significantly after four years [18].

It has been shown that increasing age and male gender has an adverse effect on the outcome of HBV immunisation [26–30]. What is noteworthy, the median age of our study participants was 52 years. Therefore, it might be expected that, in general, the immune response against HBV after vaccination with two doses in this group would be worse than in younger individuals, thus a significant proportion of them would not develop protection. In fact patients with an adequate response to HBV vaccination were significantly younger than those with an inadequate response. Furthermore, the population of patients referred for elective surgical procedures presented various co-morbidities. Although not observed in this study, possibly due to not enough numerous sub-groups of participants, some other studies have demonstrated patients in late stage kidney disease, with diabetes, chronic liver diseases and on immunotherapy are less likely to seroconvert [30].

It may be assumed that for the majority of patients vaccination means protection, no matter the number of doses taken. One point of note is that none of the study participants who preoperatively took two doses of vaccine reported being informed of the mechanisms of HBV protection via vaccination, none were asked to check anti-HBs level 1–2 months after immunization. Although Polish surgeons widely recommend a preoperative HBV vaccination with at least two doses of vaccine - they do not routinely recommend the anti-HBs testing to check if a patient has been successfully immunized. In addition, test results are not required when admitting a patient for elective surgery. In the case of a healthcare-acquired HBV infection generated during a surgical procedure, on a patient immunized with two doses schedule, the hospital may claim the patient was in the “responders” group. Thus, it is impossible to verify an infection control error made by a facility. Patient HBV immunization, even with only two doses of vaccine, allows a hospital to avoid liability regarding any untoward acquisition of a nosocomial infection. For some cases, the fear of litigation related to a nosocomial infection could be a more potent stimulus for supporting preoperative HBV immunisation than any concern regarding patient protection in the acquisition of a nosocomial HBV infection [9].

Additionally, a HBV immunisation certificate held by the patient would offer surgical staff a sense of security regarding patient-to-doctor HBV transmission. However, this also might be illusive and misleading. Data from the individual reports suggested that, in 2013, 22% of newly detected chronic hepatitis B cases in Poland received full vaccination against HBV and out of 1541 acute hepatitis B cases, six were fully vaccinated [7].

There are many areas in politics and evidence that can influence a governmental decision or other group to adopt a certain policy. The modern concept of health policy involves evidence-based policy (EBP) which relies on the use of science and rigorous studies to identify programs and practices capable of improving policy relevant outcomes [31]. There are a number of methodologies for EBP, but one of the key characteristics is to separate uncertainties and control other influences outside the policy that may have an effect on the outcome [32]. It seems that preoperative HBV vaccination obligation, in the case of elective surgery patients, in force in Poland between 1993 and 1997, was oriented more to surgical staff or hospital managers than patients.

Clearly, in the light of the results of this study, uncertainty regarding the benefits for patients of only taking a two-dose schedule, regarding protection from acquiring a nosocomial infection remains, and there is a certain amount of scepticism on whether other influences were thoroughly studied outside this vaccination policy that may have also had an effect on patients.

### Limitations

The strong point of the current study is its pioneering character. Moreover, the study was conducted among patients from the randomly selected hospitals, therefore the study population may be a good representative of the whole region.

There are several limitations in our study. Firstly, the sample size was rather small which might influenced the observed associations or not reveal existing ones. Further studies on a bigger sample would be of value. Apart from studied variables, there might be some other cofactors for unsuccessful vaccine response, e.g. genetic factors [30, 33, 34], which were not evaluated. Finally, considering a cross-sectional design, it was not possibile to rule out any cause-effect relationship between the factors assessed and vaccine response.

## Conclusions

In conclusion, the success rate in achieving adequate immune protection with two dose HBV vaccination schedule in preoperatively vaccinated patients is relatively low. Current policy regarding a preoperative 2-dose vaccination schedule in Poland should be revised. According to results presented in Table 2 and Fig. 3, the best time to perform surgery after the implementation of the second dose of HBV vaccine in the context of patient protection against HBV infection would be between 38 and 60 days. Patients should be informed about the mechanisms of HBV protection via vaccination, with an emphasis on the sero-conversion rates and determinants regarding the number of doses taken; this should be followed by signed patient consent in any medical documentation. Testing for anti-HBs level 1–2 months after taking the second dose should be highly recommended. Special attention should be paid to those vaccinated less than two months before surgery due to the highest percentage of those unprotected observed in this group. Postponing the operation over 60 days after taking the second dose may result in obtaining better protection.

In more than a third of cases the standard three-dose regimen could have been implemented, as participants had time to complete a third dose. Therefore, effective immune response should be targeted by a thorough insight in a vaccination history while referring a patient for an elective surgery, which would result in completing the third dose when there is such an opportunity. Last but not the least, to reduce the number of hospital acquired infections, facilities should promote infection prevention and control strategies which are essential [35, 36].

## Abbreviations

95%-CI: 95%-confidence interval
anti-HBc: hepatitis B core antibody
anti-HBs: hepatitis B surface antibody
EBP: Evidence-based policy
ELISA: Enzyme-linked immunosorbent assay
HB: Hepatitis B
HBs Ag: Surface antigen of the hepatitis B virus
HBV: Hepatitis B virus
OR: Odds ratio
